# Modulation of Neuronal Activity on Intercalated Neurons of Amygdala Might Underlie Anxiolytic Activity of a Standardized Extract of* Centella asiatica* ECa233

**DOI:** 10.1155/2018/3853147

**Published:** 2018-04-24

**Authors:** Aree Wanasuntronwong, Oraphan Wanakhachornkrai, Penphimon Phongphanphanee, Tadashi Isa, Boonyong Tantisira, Mayuree H. Tantisira

**Affiliations:** ^1^Department of Oral Biology, Faculty of Dentistry, Mahidol University, Bangkok 10400, Thailand; ^2^Department of Medical Sciences, Faculty of Science, Rangsit University, Pathum Thani 12000, Thailand; ^3^National Institute for Physiological Sciences, Okazaki 444-8585, Japan; ^4^Faculty of Pharmaceutical Sciences, Chulalongkorn University, Bangkok 10330, Thailand; ^5^Faculty of Pharmaceutical Sciences, Burapha University, Chonburi 20131, Thailand

## Abstract

GABAergic intercalated neurons of amygdala (ITCs) have recently been shown to be important in the suppression of fear-like behavior. Effects of ECa233 (a standardized extract of* Centella asiatica*), previously demonstrated anxiolytic activity, were then investigated on ITCs. Cluster of GABAergic neurons expressing fluorescence of GFP was identified in GAD67-GFP knock-in mice. We found that neurons of medial paracapsular ITC were GABAergic neurons exhibiting certain intrinsic electrophysiological properties similar to those demonstrated by ITC neurons at the same location in C57BL/6J mice. Therefore, we conducted experiments in both C57BL/6J mice and GAD67-GFP knock-in mice. Excitatory postsynaptic currents (EPSCs) were evoked by stimulation of the external capsule during the whole cell patch-clamp recordings from ITC neurons in brain slices. ECa233 was found to increase the EPSC peak amplitude in the ITC neurons by about 120%. The EPSCs in ITC neurons were completely abolished by the application of an AMPA receptor antagonist. Morphological assessment of the ITC neurons with biocytin demonstrated that most axons of the recorded neurons innervated the central nucleus of the amygdala (CeA). Therefore, it is highly likely that anxiolytic activity of ECa233 was mediated by increasing activation, via AMPA receptors, of excitatory synaptic input to the GABAergic ITC leading to depression of CeA neurons.

## 1. Introduction

Emotional symptoms especially fear/anxiety-like behavior are considered to be regulated through the amygdala region. The stimuli from the environment reach the basolateral amygdala (BLA) via thalamic and cortical afferents and transmit to the central nucleus of amygdala (CeA) where a proper anxiogenic response occurs [1]. Between BLA and CeA, the relay local processing happens at the intercalated cells (ITCs) level. The ITCs of the amygdala consist of densely packed GABAergic neurons surrounding BLA providing inhibitory control of neurons in BLA and CeA [2]. This group of neurons receives information from both intra- and extra-amygdaloid nuclei. The external capsule is the major connecting fiber to the ITCs. However, the exact fiber in external capsule that inputs to the ITCs is not clearly identified [3, 4]. Nevertheless, the projections from the medial prefrontal cortex to ITCs are thought to be a critical component of the forebrain circuitry for fear extinction [3]. Furthermore, ablation of ITCs has led to extinction retrieval deficits indicating the requirement of ITCs for the expression of fear extinction [5]. Thus, previous data suggest that ITCs play a critical role in fear extinction; a very important translational process since extinction-based exposure therapies is the mainstay for the treatment of anxiety disorder [6]. Therefore, it is our interest to explore how ITCs would respond to ECa233, a standardized extract of* Centella asiatica* previously shown to possess anxiolytic activity by our group [7].

*Centella asiatica *(CA), one of psychoactive medicinal plants, has been used in Ayurvedic medicine for centuries to relieve the symptom of anxiety and to contribute a deep state of relaxation and/or mental calmness throughout meditation practices. A recent clinical study showed the beneficial effects of CA on generalized anxiety disorder [8]. As to the mechanism of its action in brain function, CA is considered to enhance the GABAergic neurotransmission at the presynaptic level by facilitating the glutamic acid decarboxylase activity [9]. However, the possible function of CA on the glutamatergic synaptic transmission to GABAergic neurons remained unclear. A standardized extract of* Centella asiatica* or ECa233 was prepared to avoid the large variation in composition of herbal extracts and to ensure consistency in physical and chemical constituents. ECa233 contained triterpenoid glycosides not less than 80% and the ratio between madecassoside and asiaticoside should be 1.5 ± 0.5. Previous studies demonstrated that ECa233 exerted the neuroprotective effects on learning and memory deficit induced by 2-vessel occlusion (2VO) in mice at the doses of 10 and 30 mg/kg [10]. It protected the neuronal cell death in hippocampus. Moreover, the anxiolytic activity of ECa233 on stress-induced anxiety in chronic immobilization models was observed [7]. Interestingly, the effect of ECa233 on ERK1/2 signaling pathways was reported to promote the neurite outgrowth of neuronal cells [11].

## 2. Materials and Methods

### 2.1. Animals

The protocol of animal housing and treatment used in this study was approved by the Ethics Committee of the Faculty of Pharmaceutical Sciences, Chulalongkorn University, Thailand, and the Institutional Animal Care and Use Committee of the National Institutes of Natural Sciences, Japan. The GAD67-GFP knock-in and wild type C57BL/6J mice aged between 14 and 21 days were used for slice preparation. The procedures for the generation and genotyping of the GAD67-GFP knock-in mice were described by Kaneko et al., 2008 [12]. Mice homozygous for the GAD67-GFP allele were mated with C57BL/6J wild type mice to obtain the heterozygous mice. Wild type C57BL/6J mice were obtained from the National Laboratory Animal Center, Mahidol University.

### 2.2. Electrophysiological Studies

Male GAD67-GFP knock-in or wild type C57BL/6J mice were decapitated under deep isoflurane anesthesia. Their brains were quickly removed and submerged for 2-3 min in ice cold modified Ringer's solution containing (in mM): 234 sucrose, 2.5 KCl, 1.25 NaH_2_PO_4_, 10 MgSO_4_, 0.5 CaCl_2_, 26 NaHCO_3_, and 11 glucose and bubbled with 95% O_2_/5% CO_2_, pH7.4. Coronal slices (~300 *μ*m thickness) of the amygdala at approximately 1.64 mm caudal to bregma were cut with Microslicer (DTK-2000; Dosaka EM, Kyoto, Japan) and incubated with standard Ringer solution at room temperature for 1 h before recording. The standard Ringer solution contained (in mM) 125 NaCl, 2.5 KCl, 2 CaCl_2_, 1 MgCl_2_, 26 NaHCO_3_, 1.25 NaH_2_PO_4_, and 25 glucose and bubbled with 95% O_2_/5% CO_2_, pH 7.4. After incubation, slices to be used for recording were placed individually in a recording chamber on an upright microscope (DM LFS; Leica, Wetzlar, Germany) and continuously superfused with standard Ringer solution at a rate of 3–5 ml/min using a peristaltic pump (Minipuls 3, Gilson, Villiers, France). The tested substance, ECa233, was bath applied at the final concentration in standard Ringer solution.

The procedures of whole cell patch-clamp recordings are described previously [13]. Whole cell patch-clamp recordings were conducted on the neurons in ITC under visual control of patch pipettes using a patch-clamp amplifier (EPC-7; List, Darmstadt, Germany). GFP-positive neurons were selected using epifluorescence microscopy and then visualized with Nomarski optics with ×63 water-immersion objective lens. Patch pipettes were pulled from borosilicate glass capillaries (GC150T-10, 1.5 mm OD, Harvard Apparatus, Kent, UK) using the horizontal electrode puller (P-97; Sutter Instrument, CA, USA). The pipettes were filled with a gluconate-based intracellular solution containing (in mM): 140 K-gluconate, 20 KCl, 0.2 EGTA, 2 MgCl_2_, 2 Na_2_ATP, 0.5 Na_3_GTP, 10 HEPES, and 0.1 spermine (pH 7.3) for the study of firing pattern, passive, and active properties of neurons. The Cs-based intracellular solution was used to study the AMPA and NMDA receptor-mediated excitatory postsynaptic currents, effect of ECa233 on eEPSCs consisting of (in mM): 120 Cs-gluconate, 10 CsCl, 2 MgCl_2_, 4 Na_2_ATP, 10 EGTA, 10 HEPES, and 0.1 spermine (pH 7.3). To stain the recorded neurons, biocytin (4 mg/ml; Sigma, St. Louis, MO) was dissolved in the intracellular solution. As the liquid junction potential between the standard Ringer's solution and the gluconate-based intrapipette solution was estimated at −10 mV, the actual membrane potential was corrected by this value. The osmolarity of the intrapipette solution was 280–290 mOsm/L. The resistance of the electrodes is 5–8 MΩ in the bath solutions. All recordings were performed at 32–34°C. Data were acquired using the pClamp system (pClamp 8.1; Molecular Devices, CA, USA).

### 2.3. Comparative Electrophysiological Properties of ITCs between GAD67-GFP Knock-In and Wild Type C57BL/6J Mice

Initially electrophysiological recordings were made on GABAergic neurons of ITC, easily identified by GFP fluorescence, in GAD67-GFP knock-in mice. Depolarizing current pulses were applied with 400 ms duration and step amplitudes up to 200 pA (40 pA in each step). The resting membrane potential (RMP), input resistant (Rin), threshold, and action potential amplitude were recorded. The same procedure was applied on neurons at the same location in wild type C57BL/6J mice and similar electrophysiological characteristics were obtained indicating GABAergic properties of the recorded neuron which were further used for analysis of ECa233 activity on ITC of amygdala.

### 2.4. Effect of ECa233 on EPSCs of ITCs

**Figure 1:**
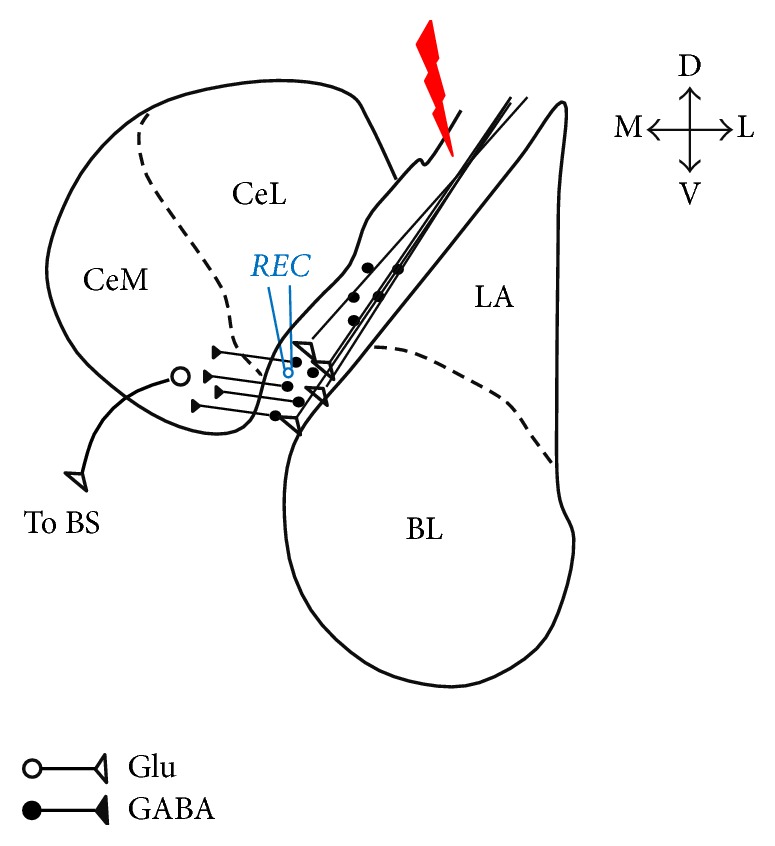
Schematic diagram of the brain slice at approximately 1.64 mm caudal to bregma level showing the location of stimulating and recording electrodes in the ITC clusters.

**Figure 2:**
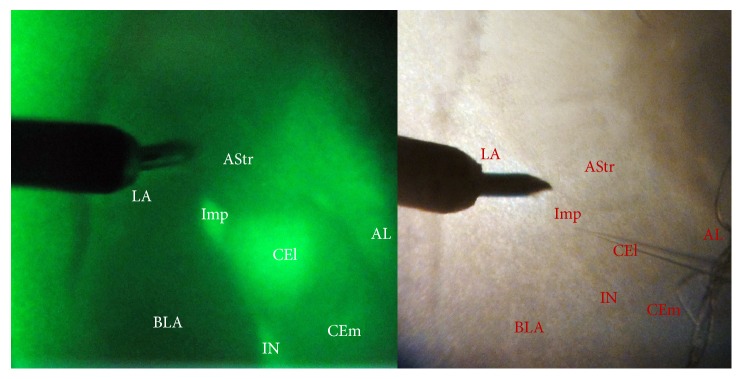
The comparison of amygdala area morphology between GAD 67-GFP knock-in and wild type C57BL/6J mice. The whole cell patch-clamp recording was made in medial paracapsular ITC (Imp) neurons. Stimulating electrode was placed in external capsule. LA, lateral amygdala; BLA, basolateral amygdala; Imp, medial paracapsular ITC; IN, main ITC nucleus; CEl, lateral subdivision of the central amygdala; CEm, medial subdivision of the central amygdala; Astr, amygdalostriatal transition area; and AL, nucleus of the ansa lenticularis.

Electrical stimulation of external capsule was applied as a cathodal square wave pulse of 200 *μ*s duration with an intensity of up to 150 *μ*A using a concentric bipolar electrode (Clark Electromedical Instruments, Pangbourne, UK) to activate the input fibers to ITCs (Figures 1 and 2). ECa233 was bath applied to examine the EPSCs on ITCs. Data was analyzed with Clampfit (Molecular Devices, CA, USA). Statistical significance was examined with a two-tailed Student's paired *t-*test and the difference was considered significant if *p* < 0.05. To visualize the recorded neurons, 4 mg/ml biocytin was dissolved in the intrapipette solution and diffused into the neuron during the patch-clamp recordings. In order to distribute biocytin throughout the recorded neuron (estimated to take 30 min), slices were fixed >30 min. The patch pipettes were carefully detached from the cells after recording. The slices were fixed with 4% paraformaldehyde in 0.12 M phosphate buffer, pH 7.4, for 2-3 days at 4°C, rinsed in 0.05 M PBS, pH 7.4, and incubated in methanol containing 0.6% H_2_O_2_ for 30 min. After washes with PBS, the slices were incubated in avidin-biotin peroxidase complex solution (1%) (Vector Laboratories, Burlingame, CA) containing 0.3% Triton X-100 for 3 h. After washes with PBS and 0.05 M Tris-buffered saline (TBS), pH 7.6, they were incubated in a TBS solution containing 0.01% diaminobenzidinetetrahydrochloride (DAB), 1% nickel ammonium sulfate, and 0.0003% H_2_O_2_ for 30 min. All procedures for visualization of biocytin were performed at room temperature. The slices were mounted on gelatin-coated slides, counterstained with cresyl violet, dehydrated, and then coverslipped. The morphological properties of stained cells were drawn using a camera lucida attached to a light microscope (Nikon, Japan).

### 2.5. Chemicals and Test Compounds

The preparation and the phytochemical profile of ECa233 were described by Wanasuntronwong et al., 2012 [7]. All drugs and chemicals in the electrophysiological study were purchased from Sigma (St. Louis, MO). We used 10 *μ*M bicuculline methobromide (Bic), 10 *μ*M 6-cyano-7-nitroquinoxaline-2,3-dione (CNQX), and 50 *μ*M D-2-amino-5-phosphonovaleric acid (AP-5) for suppression of GABA_A_ receptors, AMPA/kainite receptors, and NMDA receptors, respectively.

### 2.6. Statistical Analyses

Results are represented as mean ± SEM from eight to ten animals per group. Statistical differences of the mean were analyzed using one-way analysis of variance (ANOVA) followed by Dunnett's post hoc test for a significance level when the “*P*” value was less than 0.05. Simple comparison between two groups was performed using Student's *t*-test. All statistical analyses were performed using PASW Statistics 18 (SPSS Inc, Chicago, USA).

## 3. Results

### 3.1. The Morphological and Electrophysiological Properties of ITCs in GAD67-GFP Knock-In and Wild Type C57BL/6J Mice

**Figure 3:**
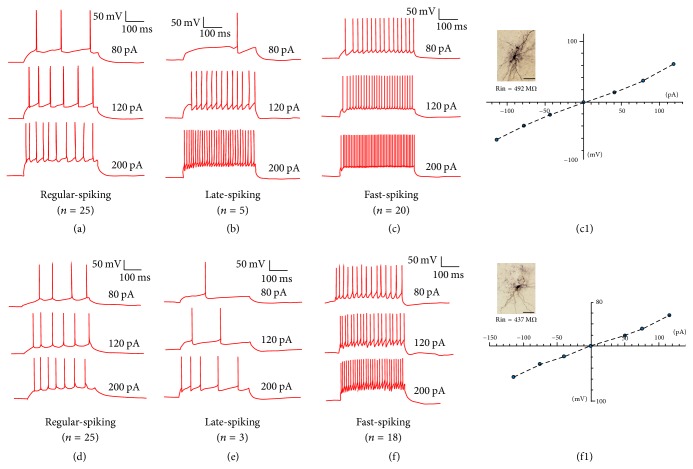
Intrinsic electrophysiological properties of ITCs. (a) (*n* = 25), (b) (*n* = 5), and (c) (*n* = 20) are the properties of ITCs in GAD67-GFP knock-in mice. (d) (*n* = 24), (e) (*n* = 3), and (f) (*n* = 18) are the properties of ITCs in C57BL/6J mice. (c1) and (f1) are the light microscopy image of a representative biocytin filled in ITC neurons (typical bipolar structure) with their input resistance with *I*-*V* plot (scale bar = 50 *μ*m).

Firstly, we use GAD67-GFP knock-in mice to identify the GABAergic neurons in the ITCs. We found that ITC was a well-established structure with clear boundaries and almost all were GABAergic neurons. Comparative structure between GAD67-GFP knock-in and wild type C57BL/6J mice was shown in Figure 2. The boundary of this area was also visible in wild type C57BL/6J mice. Electrical properties of ITC neurons in GAD67-GFP knock-in and wild type C57BL/6J mice were investigated. The firing pattern and morphological properties of neuron in ITC between GAD67-GFP knock-in and wild type C57BL/6J mice showed no differences (Figure 3). Three types of firing pattern were demonstrated: regular spiking, late spiking, and fast spiking. The morphological properties were typically bipolar with the I-V plots of input resistance (Figures 3(c1) and 3(f1)). The passive and active membrane properties of neurons in both mice showed no difference (Table 1). The *P* value of RMP, Rin, threshold, and action potential amplitude was 0.195, 0.832, 0.565, and 0.586, respectively, by Student's pair *t*-test.

### 3.2. AMPA/NMDA Receptors Mediated eEPSCs Recorded in the ITC

**Figure 4:**
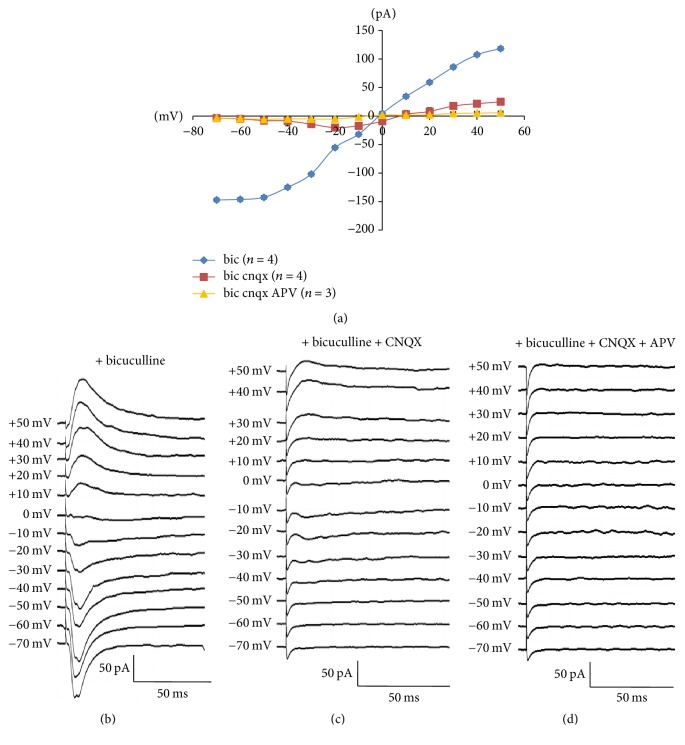
Ionotropic glutamate receptors mediating electrically evoked EPSCs (eEPSCs) recorded in the ITCs. Voltage clamp recording after ITC stimulation at *V*_*H*_ (voltage holding) ranging from −70 mV to +50 mV. (a) Summary plot of the peak amplitude of glutamate, NMDA, and nonglutamate receptor-mediated eEPSCs against *V*_*H*_. (b) Glutamate receptor-mediated synaptic currents in the presence of bicuculline (10 *μ*M). (c) NMDA receptor-mediated eEPSCs in the presence of bicuculline (10 *μ*M) and CNQX (50 *μ*M). (d) Nonglutamate receptor-mediated eEPSCs in the presence of bicuculline (10 *μ*M), CNQX (50 *μ*M), and APV (10 *μ*M).

In this study, the stimulating electrode was placed on the external capsule and the recording electrode was patched on neurons in the ITCs cluster (Figures 1 and 2). In order to examine the voltage dependence of the evoked EPSCs (eEPSCs), recording was performed in the presence of bicuculline (10 *μ*M) to block GABA_A_ receptor activity and the Cs-based intracellular patch solution was used to reduce outward potassium current and block GABA_B_ receptors. The ionotropic glutamate receptor-mediated component at these synapses had a linear current-voltage (*I*-*V*) relationship and reversal potential of +1.2 mV (Figures 4(a) and 4(b)). NMDA-EPSCs were isolated by blocking AMPA/kainate receptor with CNQX (50 *μ*M) in the presence of bicuculline. The NMDA receptor-mediated component at these synapses had a nonlinear *I*-*V* relationship, with a region of negative slope between −40 and +10 mV and reversal potential of 7.8 mV (Figures 4(a) and 4(c)). Additionally, the application of 10 *μ*M APV (a selective NMDA receptor antagonist) in the presence of bicuculline and CNQX totally abolished the response (Figures 4(a) and 4(d)).

### 3.3. ECa233 Increased the Amplitude of eEPSCs in ITC

**Figure 5:**
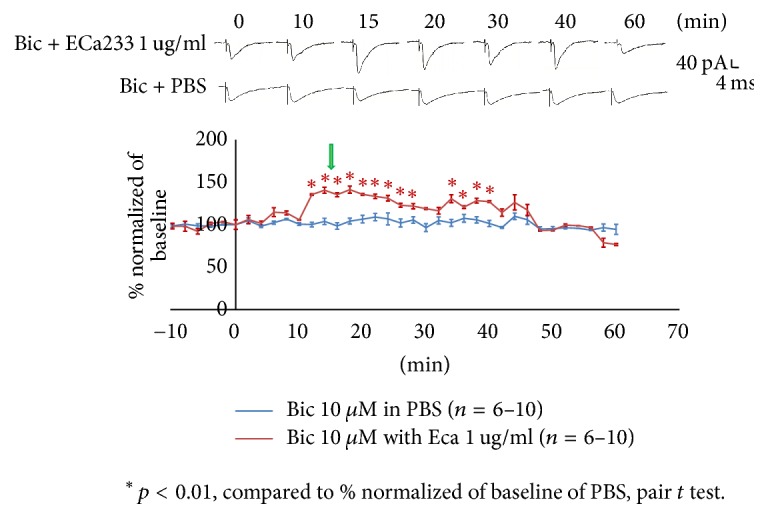
Effect of ECa233 on EPSCs in ITCs recorded in every 2 min for 1 h. The maximal response was noted approximately at 15 min. Tracing showed the example of EPSCs at 0, 10, 15, 20, 30, 40, and 60 min after ECa233 1 *μ*g/ml with bicuculline (10 *μ*M) bath application compared with the PBS in the presence of bicuculline. The EPSCs in the presence of ECa233 1 *μ*g/ml were normalized with respect to control condition (*n* = 5–10, ^*∗*^*P* < 0.01; pair *t*-test).

To study the effect of ECa233 on the eEPSCs, we continuously recorded the eEPSCs in ITC neurons every 2 min for 1 h. We found that the eEPSCs gradually increased 10 min after initiation of bath application of ECa233 (Figure 5). The maximal response was noted approximately at 15 min. Upper traces showed the examples of the eEPSCs recorded at 0, 10, 15, 20, 30, 40, and 60 min after bath application of 1 *μ*g/ml ECa233 compared with the application of PBS. All of the solutions contained bicuculline 10 *μ*M.

### 3.4. Concentration-Response Curve of ECa233

**Figure 6:**
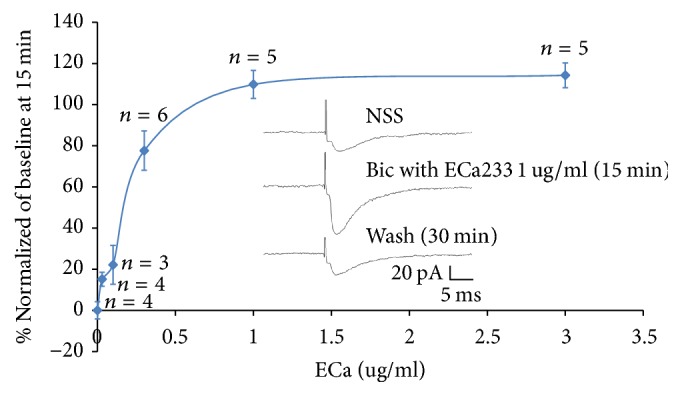
Concentration-response curve for the enhancement of EPSCs of ITC by ECa233. The EPSCs in the presence of ECa233 and bicuculline were normalized with respect to the control condition and were averaged (each point was the mean ± SEM of 5–8 neurons). Tracing showed before and after 15 min application of ECa233 and 30 minutes PBS washes the effect of ECa233.

To characterize the excitatory effects of ECa233 on ITC neurons, the concentration-response curve of ECa233 was obtained by testing the effects of different concentrations of ECa233 (Figure 6). ECa233 (0, 0.03, 0.1, 0.3, 1.0, and 3.0 *μ*g/m) generated a concentration-dependent enhancement of EPSCs in ITC neurons. The estimated maximal increase of eEPSCs was 120%, and the estimated value of EC_50_ was 0.25 *μ*g/ml. Effects of ECa233 were completely abolished after being washed by PBS for 30 min. Example tracing of eEPSCs before and after a bath application of ECa233 (1 *μ*g/ml) for 15 min was shown in the inset of Figure 6. All of the solutions contained 10 *μ*M bicuculline.

### 3.5. Effect of ECa233 in the Presence of Blockers of Fast Synaptic Transmission

**Figure 7:**
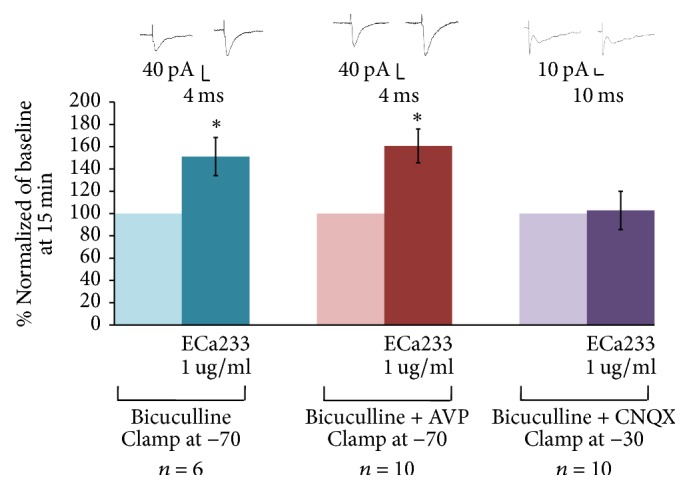
ECa233 increases EPSCs in ITC by mediating through the ionotropic glutamate receptor. The data were normalized to the control condition in the presence of bicuculline (*n* = 6; ^*∗*^*P* < 0.05, paired *t*-test) or bicuculline + APV (*n* = 10; ^*∗*^*P* < 0.05, paired *t*-test), or bicuculline + CNQX.

We examined the ECa233-induced increase of eEPSC amplitudes in ITC neurons in the presence of various blockers of synaptic transmission. Bicuculline, a GABA_A_ receptor antagonist, was used to exclude the interference of IPSCs. In the presence of bicuculline, 1 *μ*g/ml ECa233 enhanced the eEPSCs in ITCs to 151.19 ± 17.11% suggesting that the enhancement was mediated via fast synaptic glutamatergic transmission. To determine whether the excitatory effect of ECa233 on ITC neurons was mediated by AMPA or NMDA receptors, eEPSCs were recorded in the presence of bicuculline + CNQX or bicuculline + APV. It was found that bicuculline + APV failed to block the effect of ECa233 (160.69 ± 15.23%) on eEPSCs, whereas bicuculline + CNQX completely abolished this effect (102.81 ± 17.16%), suggesting involvement of AMPA/kainate receptors in the facilitation of eEPSCs in ITC neurons by ECa233 (Figure 7).

### 3.6. Postsynaptic Targets of ITC Neurons

**Figure 8:**
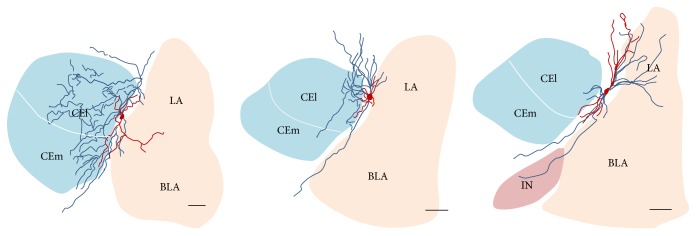
Camera lucida drawings of ITC neurons sending the collateral axons to several brain areas mostly into the CA. Scale bar = 100 *μ*m.

We examined further the postsynaptic targets of biocytin filled ITC axon terminal. The result showed that ITC neurons had medium ovoid cell bodies, largely bipolar dendritic trees, and axons that send collaterals into several areas such as LA, BLA, ITC, main nucleus of ITC (IN), and amygdalostriatal transition area (Astr) and mostly in CeA (Figure 8).

## 4. Discussion

In the present study, bath application of ECa233 significantly increased evoked EPSCs of ITCs up to 120%. Further experiments conducted in the presence of a series of antagonist(s) suggested that modulation of AMPA receptors was responsible for the increased evoked EPSPs observed and ultimately leading to anxiolytic activity in animal models. To our knowledge, this is the first report to demonstrate a modulation of ITCs of amygdala as possible mechanism underlying anxiolytic activity of a natural product, the standardized extract of* Centella asiatica *ECa233 in animal models. Furthermore, our findings could possibly suggest the prominent role played by ITCs in fear extinction as a potential pharmacological treatment of anxiety as well.

Due to the lack of supply of GAD67-GFP knock-in mice in Thailand and our findings that the morphological and electrical properties including active and passive membrane properties of specific cluster of neurons in GAD67-GFP knock-in and C57BL/6J mice were not different [14, 15], we conducted experiments using C57BL/6J mice to study the effect of ECa233, a natural extract of* Centella asiatica,* on ITCs neurons.

Stimulation of ITCs was made by a concentric bipolar electrode placing on external capsule encompassing a number of fibers from various nuclei including mPFC-ITCs pathway responsible for anxiolytic-like behavior seen in elevated plus maze and open field test [7]. Recently, there were numerous studies of fear extinction-based therapies for the treatment of anxiety disorder focusing on the mPFC-ITCs pathway. The density of mPFC fibers innervated the ITCs is a subject of controversy. Several laboratories indicated that the mPFC provides a direct innervation to ITCs [3, 16]. However, Strobel and their coworker showed the di-synaptic of mPFC-ITCs via the BLA [4]. Therefore, at the moment, anxiolytic activity of ECa233 acting on ITCs may or may not involve mPFC-ITCs pathway. Synaptic inputs of ITC neurons coming from the external capsule are glutamatergic synapses similar to the result of main intercalated cells [14], suggesting that long term depression and potentiation can occur in the ITCs. It is well known that CeA promotes the anxiogenic-like behaviors. For example, the injection of muscimol was found to impair the avoidance of rats to the open arms of the elevated plus maze [17]. So, the strongest feed forward inhibition of ITCs to CeA should present the anxiolytic activity. ECa233 increased the eEPSCs in ITCs via the AMPA receptors as mentioned in this experiment. The finding that brain slices had to be perfused with ECa233 in 5–10 min before the effects were detected suggested the possibility that ECa233 might increase the phosphorylation of AMPA receptor. Previous study of our group demonstrated the upregulation of ERK1/2 and PI3K/Akt signaling pathways after ECa233 exposure [11]. Increased PI3K/AKT signaling was associated with increased expression of AMPA receptors in the surface membrane [18]. In addition, superfusion of ECa233 for 10 min in brain slice preparation increased the long-term potentiation and the BDNF protein expression in hippocampus (unpublished observation). Unfortunately, in the present study, no attempt is made to probe for a presynaptic or postsynaptic site of action for ECa233. This topic should be further investigated in the future.

In line with other studies, the collateral axon of ITCs in the present study mainly innervates CeA [2, 15] suggesting that facilitation of ITCs activity by ECa233 could therefore lead to an increased GABAergic inhibition of CeA. GABA is known as major inhibitory neurotransmitter of the brain and dysfunction of GABAergic neurotransmission causes neurological disorder. Thus, activities of ECa233 on other neuronal circuitry using GABA are plausible and await proof of evidence.

Taken all together, it is highly likely that ECa233 modulated the AMPA receptor and consequently activated ITC neuron to release GABA neurotransmitter in CeA, which then suppressed the CeA neuronal activity. Thus, it is possible that ECa233 acutely reduced the anxiety behavioral responses in animal models by increasing the GABA release in CeA via modulation of ITC neurons. Similar results were previously reported on neuregulin-1, the neurotrophic factor that could u-regulate AMPA receptor in GABAergic neurons and hence explained its benefit in the treatment of mental disorder [19].

## 5. Conclusions

The present study demonstrated the mechanism of anxiolytic activity of ECa233, a standardized extract of* Centella asiatica*, on ITCs of the amygdala. ECa233 facilitates the AMPA receptor-mediated excitatory synaptic inputs in ITCs. Since most of the ITC axons innervated CeA. We suggest that increasing the excitatory responses of ITCs by ECa233 would promote the GABA release in the CeA suppressing the output signals from the amygdala.

## Figures and Tables

**Table 1:** Comparison of electrophysiological properties between ITC in GAD67-GFP knock-in and ITC in C57BL/6J mice.

Parameter	ITC in GAD67-GFP	*n*	ITC in C57BL/6J	*n*	*P* value (*t*-test)
Resting membrane potential (mV)	−71.16 ± 4.10	18	−69.16 ± 4.66	18	*P* = 0.195
Input resistant (MΩ)	529.00 ± 59.18	18	534.33 ± 67.18	18	*P* = 0.822
Threshold (mV)	−41.41 ± 3.02	18	−40.70 ± 3.44	18	*P* = 0.565
Action potential amplitude (mV)	77.2339 ± 5.29	18	76.47 ± 3.76	18	*P* = 0.586
